# Biological activities and biosorption potential of red algae (*Corallina officinalis*) to remove toxic malachite green dye

**DOI:** 10.1038/s41598-023-40667-8

**Published:** 2023-08-24

**Authors:** Elen Emad Youssef, Botros Y. Beshay, Kareem Tonbol, Sarah O. Makled

**Affiliations:** 1 College of Pharmacy, Arab Academy for Science, Technology and Maritime Transport, Abu-Qir, Alexandria, Egypt; 2 College of Maritime Transport and Technology, Arab Academy for Science, Technology and Maritime Transport, Abu-Qir, Alexandria, Egypt; 3 Oceanography Department, Faculty of Science, Alexandria University, Alexandria, Egypt

**Keywords:** Biological techniques, Cancer, Microbiology, Environmental sciences, Risk factors, Chemistry

## Abstract

This research aims to use eco-friendly *Corallina officinalis* as an adsorbent for removing harmful malachite green dye streams from industrial effluent, promoting sustainable living and effective microbial growth inhibition. *Corallina officinalis* biomass was tested for textile dye biosorption, as well as its antibacterial, antioxidant, and cytotoxic properties. The effects of certain parameters, involving pH solution, initial dye concentration, algae dose, and contact time, were investigated on the sorption of dye. Fourier transform infrared spectroscopy and scanning electron microscopy were also used and, the results showed that the functional groups on the surface of algae played an important part in the biosorption process. It was noted that the kinetic data were significantly prominent by the Pseudo-second-order model with regression correlation coefficient ${r}_{2}^{2}$ values with an average of 0.95232. The biosorption was compatible with both the Freundlich (R^2^ = 0.9843), and Langmuir (R^2^ = 0.9653) isotherms, and the maximum removal efficiency for dye reached up to 99.9% in 2 h, 27 °C, stirring speed 120 rpm, pH 6, initial dye concentration 20 mg L^−1^, and biomass dose 0.03 g L^−1^. *Corallina officinalis* had higher antimicrobial activity, with values of minimum inhibitory concentrations ranging from 0.156 to 5 mg mL^−1^. *Corallina officinalis* exerted significant radical scavenging activity against tested free radicals. The extract was examined for cytotoxic activity using nine cancer cell lines, which exhibited high cytotoxicity for colon adenocarcinoma with an IC_50_ value of 25.895 µg mL^−1^.

## Introduction

The economic aspects of water quality and the natural resources of reusable waterways are also impacted by water pollution. Water quality control, water pollution reduction, and protection of the environment are the main concerns that need to be addressed to secure our future. Spreading infectious diseases reduces a country's chances for sustainable growth and threatens public health^1^. Numerous industries use synthetic dyes extensively to colour products such as textiles, paper, leather tanning, plastic, food, polymers, and printing. Untreated wastewater discharge is the leading cause of widespread pollution of surface and groundwater resources due to the increased toxicity and chemical oxygen demand of the effluent, as well as reduced light penetration^2^. The primary goal of industrial wastewater treatment is to safeguard human health and the environment.

An organic dye, known as Malachite Green (MG), is used in the textile industry to colour leather, silk, wool, jute, pottery, cotton, acrylic fibers, and paper. MG is a carcinogenic, mutagenic, and teratogenic substance; it has been identified as a liver tumor promoter^3^. Mutagenic, poisonous, allergic, carcinogenic, and non-degradable industrial effluents such as MG dye cause major concerns. To decrease the detrimental impacts of organically polluted wastewater on individuals and the environment, wastewater must be thoroughly cleaned before discharge into major watercourses^4^.

The removal of colours from industrial effluent is currently receiving a lot of interest. Several physicochemical techniques, including coagulation-flocculation, oxidation, membrane filtration, and adsorption, have been used to remove these industrial contaminants from liquid waste. Conventional wastewater treatment and pollutant removal from aqueous solutions are unsuccessful, and complex techniques are too expensive. As a result, there is a need to investigate novel methods whose efficiency and cost would be fascinating^5^.

Science-based policies are required to support sustainable global development as the world's population grows. It is crucial to protect the environment, restore it, and assist human society in overcoming the dangers of industrialization and unsustainable exponential expansion^6^. Within this context, many studies have shown that macroalgae are an important marine resource for ecological and sustainable living, aiding in the resolution of today's global issues such as water pollution, ocean acidification, and global warming.

Macroalgae are one of the most common photosynthetic organisms on the planet, and they can grow and survive in a variety of environments under difficult circumstances, according to Azam et al.^7^ algae are considered a promising source of biosorbents due to their high biosorption capacity and easy availability. There is considerable interest in employing algae as potential adsorption agents for treating wastewater.

The presence of sulphated polysaccharides in macroalgae cellular walls, particularly in the fibril matrix and intercellular gaps, is the primary explanation for their great capacity to bind contaminants. The polysaccharide hydroxyl, sulphate, and carboxyl groups are powerful ion exchangers^8^. Macroalgae contain special characteristics like antioxidant, antibacterial, and antitumor due to their abundance in several bioactive substances, and antifungal. Some primary and secondary metabolites such as phytochromes are among these bioactive substances (lutein and carotenoids), DHA, tannins, peptides, lipids, enzymes, vitamins, terpenoids, and other substances^9^. Macroalgae offer eco-friendly, nutritious food sources and natural components for various industries.

Motivated by these findings, we examined the removal of MG using dried *Corallina officinalis* as a low-cost adsorbent collected along the Red Sea coast in the current investigation. In this regard, the adsorption kinetics and isotherms have been investigated. The acetone extract of *Corallina officinalis* was investigated for its antioxidant, antibacterial, and cytotoxic activities, and based on study findings, we attempt to connect and explore how macroalgae can assist us in attaining three of the seventeen Sustainable Development Goals, as well as how they can promote a more sustainable way of living in the future.

## Results and discussion

### Genomic isolation and purification

**Figure 1:**
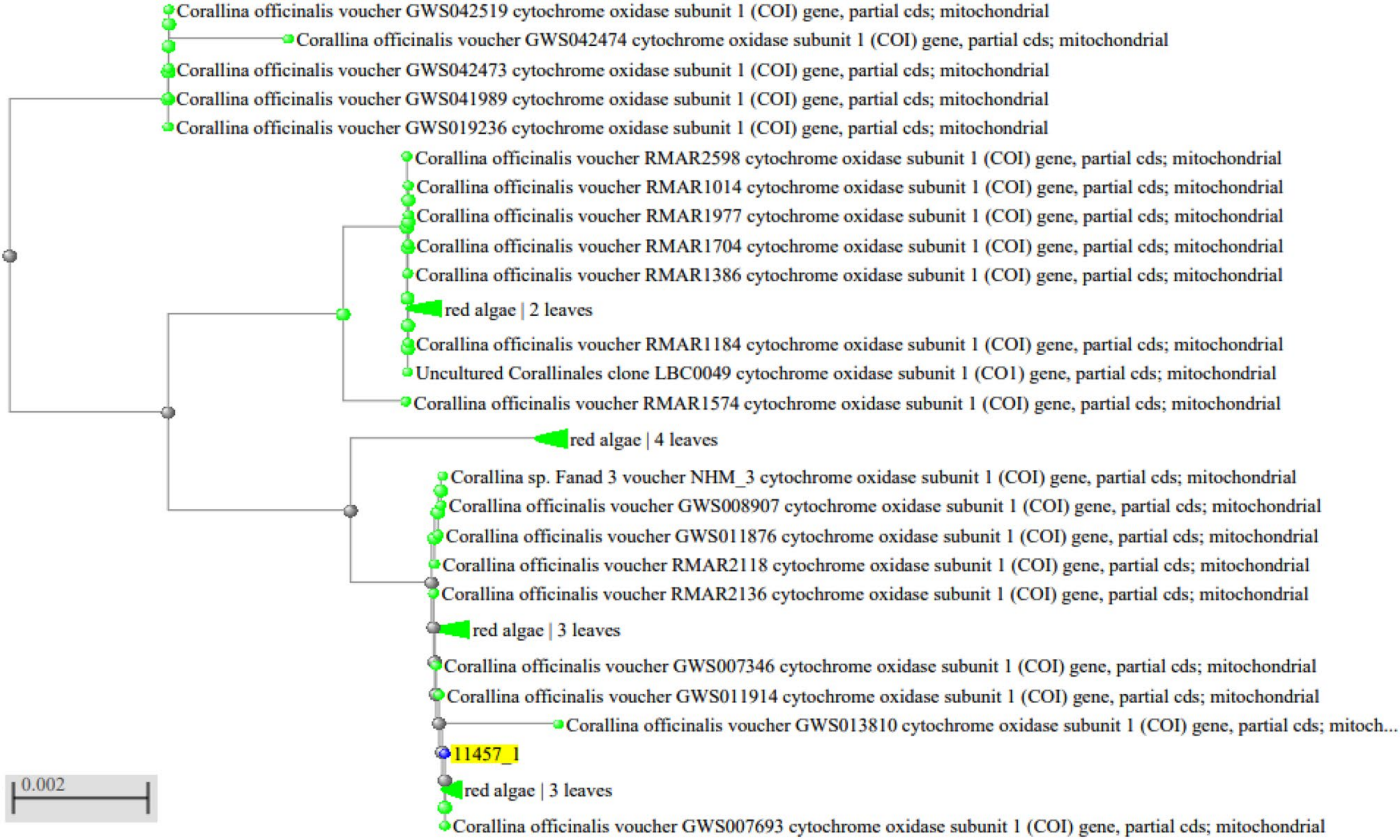
Phylogenetic tree for algae sample based on COI sequences.

Marine macroalgae are difficult to identify due to their simple morphology, convergence, phenotypic plasticity, and heteromorphic generations. It is thus not surprising that algal systematists have come to rely heavily on genetic tools^10^. Coralline samples were sequenced for the cytochrome oxidase subunit 1 (COI gene). Coralline samples from Egypt were sequenced for the COI gene. The length of determined sequences ranged from 624 to 664 bp. In the phylogenetic tree (Fig. 1), *GWS002371* was identified as *Corallina officinalis,* which formed a well-supported clade (99.84% bootstrap support) (Genbank accession: KU501319.1) (*Corallina officinalis* voucher GWS002371 (COI) gene, partial cds; mitochondrial)*.*

### IR spectral analysis

**Figure 2:**
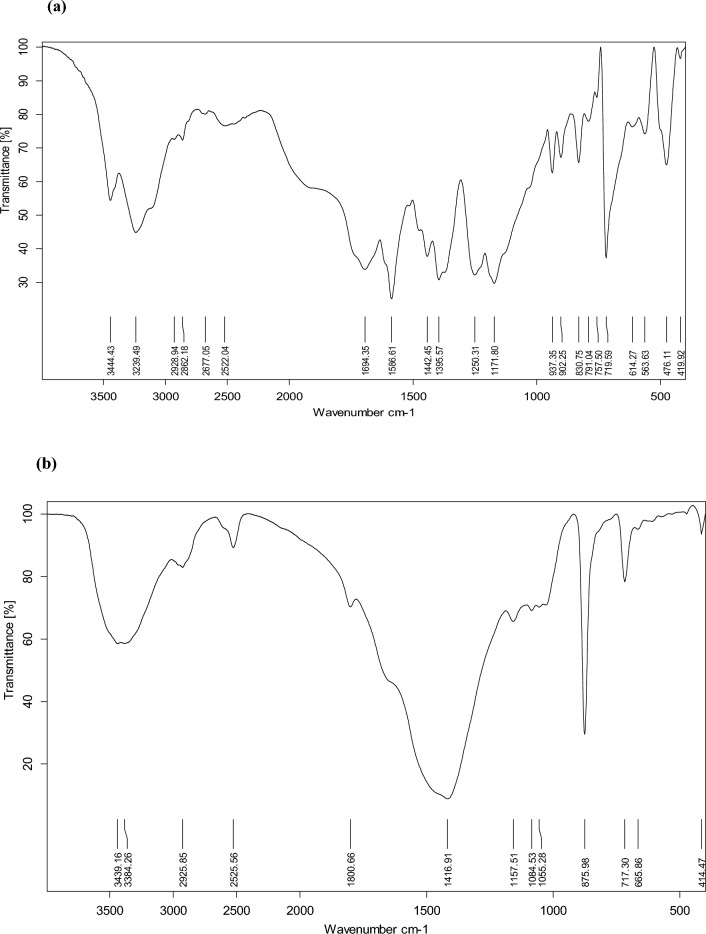
FTIR peaks of transmittance of MG (**a**) before biosorption and (**b**) MG after biosorption.

As shown in (Fig. 2), the results of the noticed IR spectra analysis before (Fig. 2a) and after (Fig. 2b) MG biosorption. Based on their wave numbers, the peaks in the FTIR spectrum were attributed to distinct functional groups at 3444–3239 cm^−1^ (O–H and N–H), 2960–2920 cm^−1^ (C–H), 2200–2552 cm^−1^ (carboxylic group), 1800–1417 cm^−1^ (amide, carboxylates, sulfates, and ketone groups), 1155–1051 cm^−1^ (C–O, C–C, and C–OH), and 876–414 cm^–1^ are associated with the aromatic groups (–C–H). Figure 2a, b illustrate some peaks change or disappear due to the binding process that occurs on the biomass's surface.

IR spectrum analysis shows that the positions of functional groups in algal biomasses alter after dye treatment, indicating that these groups are involved in MG removal. The binding process that takes place on the surface of the biomass is what causes some peaks to shift or disappear, in addition to new ones emerging upon the biosorption of MG molecules. There are many polysaccharides in the algal cell walls, some of which are linked to proteins and other elements^11^. Many different functional groups, including carboxyl, amino, and phosphate groups, are present in these molecules on the algal cell surface. It is assumed from the results that the dye interacted with the active functional groups to become part of the adsorbent as suggested by Jayaraj et al.^12^.

### Scanning electron microscopy

**Figure 3:**
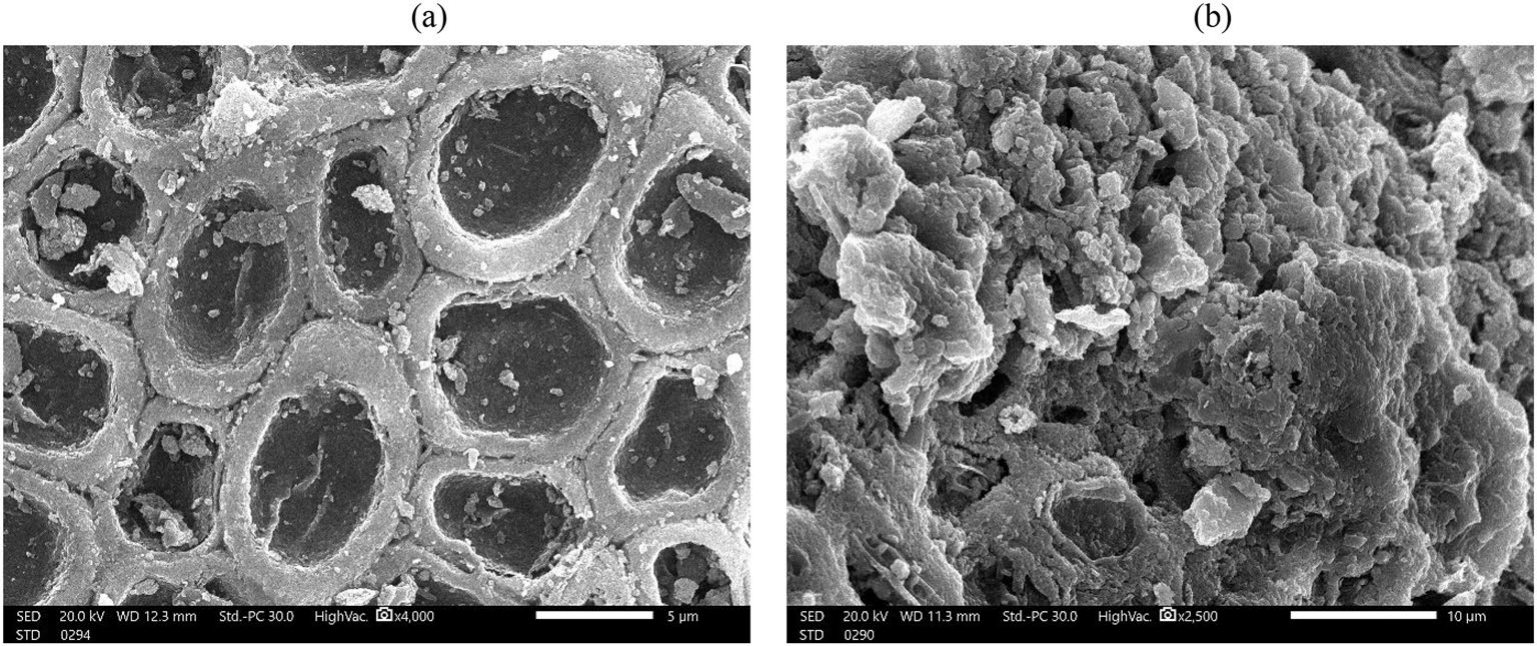
SEM images of *Corallina officinalis* (**a**) before MG biosorption and (**b**) after MG biosorption.

SEM was used to examine the morphological changes that have been brought on by dye adsorption in the cells of *Corallina officinalis* biomass. As shown in Fig. 3, there is a clear difference in the surface of the *Corallina officinalis* in before (Fig. 3a) and after (Fig. 3b) the adsorption of MG. The cells of algae appeared with highly porous deep cavities (Fig. 3a). After the adsorption of MG, the surface became flowing because of the precipitation of dye ions (Fig. 3b). A unique feature of algae seen in SEM images is the deep pores and cavities adjacent to each other. Based on the surface area to volume ratio, this wide void surface creates a large area for MG ions and increases the biosorption capacity. The surface structure and functional groups of the marine algae cell wall play a vital role in the bioadsorption capacity of algae dyes^13^. Deokar (2016)^14^ and Fakhry (2013)^15^ observed alterations in the surface porosity of algae as a result of dye adsorption.

### Biosorption experiments

**Figure 4:**
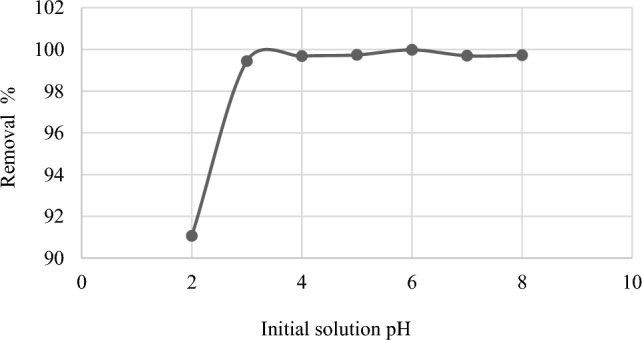
Effect of pH on the removal efficiency of MG, V: 30 mL; C_i_: 50 mg L^−1^; M: 0.03 g; Temp: 27 °C.

One of the most critical parameters affecting dye uptake from wastewater and aqueous solutions is pH. The effect of pH on dye adsorption on *C. officinalis* was investigated at 27 °C by altering the pH of the dye solution from 2 to 8. As shown in (Fig. 4), when the solution pH was raised, MG biosorption effectiveness increased. The removal efficiency % of MG reaches a maximum (99.98%) at pH 6.

pH is a critical element in dye biosorption studies because it affects the functional groups on the surface of the biomass and regulates the solubility of the dye in aqueous solutions. Because the concentration of OH^-^ increases as the pH of the solution increases, the electrostatic interaction between the negatively charged adsorbent surface and the MG molecules of cationic nature causes the adsorption capacity to increase. Previous research found that cationic dye adsorption increases with increasing pH^16^. Adsorption is reduced when the algal surface is positively charged at lower pH due to electrostatic repulsion between the cationic MG molecules and the positively charged adsorbent surface^17^.

**Figure 5:**
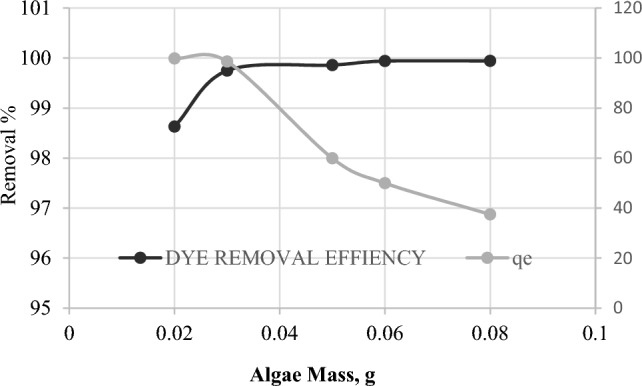
Effect of biomass on removal of MG V: 30 mL; Ci: 100 mg L^−1^; Temp: 27 °C; pH: 6.

Another key factor that determines biosorption performance is the biosorbent dose. It assesses the accessibility of binding sites for removing dye molecules at a given dye concentration. The effect of *C. officinalis* algal doses ranged from 0.02 to 0.08 g in 100 mg L^−1^ initial dye concentration at optimum pH 6, for 10 h, and at constant temperature 27 °C was investigated. Figure 5 shows that as the adsorbent mass is increased from 0.02 to 0.08 g L^−1^, the adsorption capacity (qe) declines from 99.86 to 37.48 mg g^−1^ and the percentage adsorption rises. As a result, there is an increase in the quantity of adsorption. As is well known, there are more accessible adsorption sites as the adsorbent mass increases. Furthermore, the decline in adsorption capacity with rising *C. officinalis* mass is explained by the presence of unsaturated adsorption sites^17^. This is because the active sites could be effectively utilised when the dosage was low (i.e., when the adsorbent/adsorbate ratio was low). When the adsorbent dosage is increased (high adsorbent/adsorbate ratio), it is more likely that a considerable amount of the available adsorbent will be consumed. Active areas are still uncovered, resulting in decreased specific uptake^18^. The current observations are consistent with earlier findings. Deokar et al.^14^ discovered similar qe patterns when studying several adsorbent–adsorbate systems.

**Figure 6:**
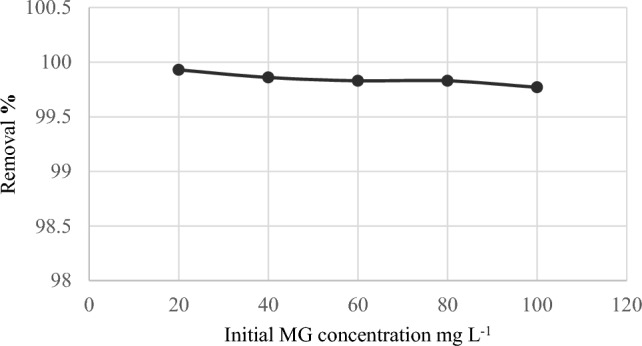
Removal of different MG concentrations by *Corallina officinalis* V: 30 mL; M: 0.03 g; Temp: 27 °C; pH: 6.

The effect of initial dye concentrations (20–100 mg L^−1^) on removal of MG by *C. officinalis* was represented in (Fig. 6) at the optimum pH 6, temperature 27 °C, biomass weight 0.03 g L^−1^, for 10 h, and at a speed of 120 rpm. The percentage of dye removed by Corallina alga slightly decreased as the dye concentration increased. Any initial concentration had the same pattern in the rate of MG biosorption. This discovery (decrease in dye % removal) is owing to the fact that all adsorbents have a finite number of active sites, which become saturated at a particular concentration^19^. The maximum dye removal rate tested was 99.93% of MG dye at the concentration of 20 mg L^−1^. The initial concentration of MG acts as the main driving factor for overcoming any mass transfer resistance between the aqueous and solid phases^20^. The rate-limiting element that affects the dye's equilibrium concentration is the availability of adsorption sites^21^, which shows the MG dye has a strong affinity for* C. officinalis*. The current results are consistent with those of Tsai et al.^19^ when studying the removal of MG from aqueous solutions by using chlorella- based biomass.

**Figure 7:**
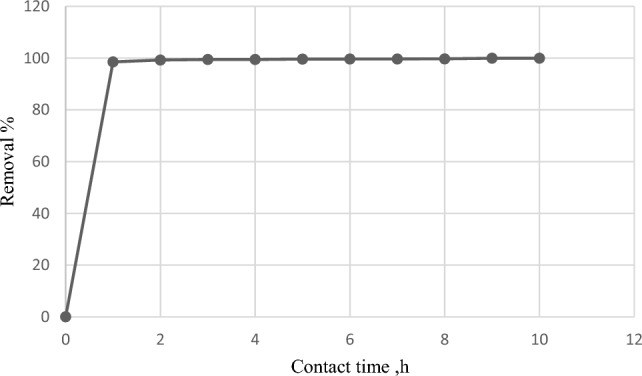
Effect of contact time on MG by *Corallina officinalis* V: 30 mL; M: 0.03 g; C_i:_ 20 mg L^−1^; pH: 6; Temp: 27°C.

Figure 7 depicts the impact of contact time (0.0–10 h) between the algal biomass and MG dye on removal effectiveness. Removing MG dye by *C. officinalis* increased quickly during the first hour at 98.51%, and then it increased gradually at 2h to 99.25%, until equilibrium. The MG biosorption process consists of three phases: rapid, slow, and stationary, with the initial fast phase due to a wide surface area and vacant macro-pores, the subsequent deceleration due to saturation, and the equilibrium in the third phase as *C. officinalis* particles were saturated^22^. The findings indicate that the functional groups on the surface wall of algae and the MG dye have a high affinity and a strong electrostatic force of attraction^11^.

Adsorption kinetics explain how rapidly the sorption process takes place. The two simplest kinetic models, pseudo-first and pseudo-second-order models were used to assess the sorption data in this study, and are explained below:

## Pseudo-First Order

This model suggests that the variation in dye concentration with respect to time is proportional to the power one. It is also predicated on the assumption that the following reaction rate r can be expressed as:

1 $$-r =-\frac{dc}{dt}=\mathrm{ k}1C$$

Separating and integrating Eq. (1) with respect to the limits *C* = *C*_0_ at *t* = 0 and *C* = *C* at any *t* yields

2 $$-{\int }_{{C}_{0}}^{c}\frac{dC}{C}={K}_{1}{\int }_{0}^{t}dt$$

3 $$\mathrm{Or\, ln}\frac{C}{{C}_{0}}=- {K}_{1} t$$

**Figure 8:**
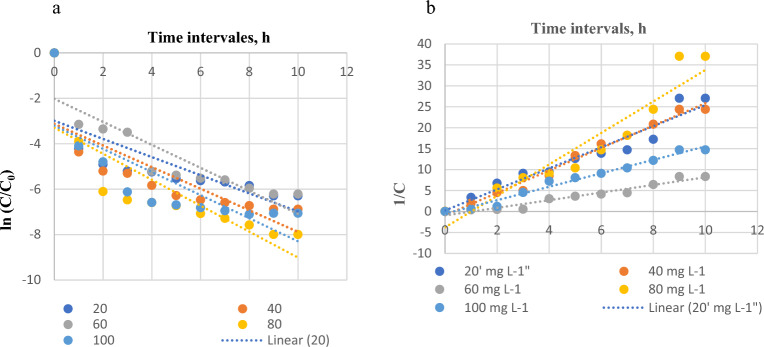
(**a**) Pseudo-first order graph; (**b**) pseudo-second order graph for the biosorption of MG dye onto *Corallina officinalis* biosorbent.

The rate constant K can thus be calculated from the slope of the plot of ln*(C/C*_*0*_*)* versus *t*. Figure 8a depicts a plot of ln(*C*/*C*_0_) versus *t* at five different dye concentrations. Table 1 displays the calculated K_1_ values and their corresponding linear regression correlation coefficient ${r}_{1}^{2}$ values. The first-order kinetic model failed to adequately represent the sorption data, with an average correlation coefficient of 0.52548.

**Table 1:** Kinetic constants for MG biosorption onto *C. officinalis*.

C_0_ (mg L^−1^)	K_1_ (${\mathrm{min}}^{-1}$)	${\mathrm{r}}_{1}^{2}$	K_2_ (L mg^−1^ s^−1^)	${\mathrm{r}}_{2}^{2}$
20	0.0222	0.5676	0.0007	0.9783
40	0.0239	0.6236	0.0006	0.9371
60	0.0184	0.7845	0.0002	0.9461
80	0.0257	0.6597	0.0007	0.9158
100	0.0243	0.6156	0.0003	0.9783

### Pseudo-second order

The sorption kinetics were further investigated with a second-order model. This model is predicated based on the assumption that the following reaction will occur:

4 $$2\mathrm{A}\longrightarrow \text{products}$$

where A is the dye component that is accumulating on the solid adsorbent the reaction rate r that can be written as:

5 $$-\mathrm{r}=-\frac{dC}{\mathrm{d}t}={K}_{2}{C}^{2}$$

When Eq. (5) is integrated with respect to the limit *C* $=$ *C*_*0*_ at time *t* $=$ 0 and *C* $=$ *C* at any time *t*, the equation simplifies to

6 $$\frac{1}{C}=\frac{1}{C0}+{K}_{2}t$$

The second-order rate constant K_2_ can be calculated using the slope of the 1/C versus t (Fig. 8b). Table 1 displays the calculated second-order rate constants K_2_ and their corresponding higher linear regression correlation coefficient ${r}_{2}^{2}$ values with an average of 0.95232. At all dye concentrations, larger ${r}_{2}^{2}$ values were found to be higher than ${r}_{1}^{2}$ value, which confirmed that the MG biosorption kinetics follows the pseudo-second-order model. In order to choose the ideal operating conditions for the large-scale batch operation, the kinetics of solute uptake must be understood. Determining the time dependency of adsorption systems under various process conditions is also critical^13^. The first-order kinetic model was unable to accurately reflect the sorption data at any of the initial dye concentrations, with an average correlation coefficient of 0.52548. Table 1 displays the determined second-order rate constants K_2_ and their correspondence to a higher linear regression correlation coefficient ${r}_{2}^{2}$ values with an average of 0.95232. At all dye concentrations, larger ${r}_{2}^{2}$ values were found to be higher than ${r}_{1}^{2}$ value confirmed that the MG biosorption kinetics follows the pseudo-second order model. So, the kinetics data were significantly represented by the pseudo-second order. According to Table 1, MG biosorption may occur in a monolayer on the surface of the adsorbent. Chemical sorption is thought to be the rate-limiting step according to the second-order kinetic model^23^.

*Corallina officinalis* is an efficient, cost-effective biosorbent for removing hazardous malachite green dye from aqueous solutions, offering a feasible alternative for water treatment. *C. officinalis* is an eco-friendly biosorbent for water treatment, offering a sustainable alternative to synthetic chemicals. It effectively removes malachite green dye with a maximum adsorption capacity of 101.30 mg g^-1^, demonstrating its ability to capture and eliminate dye. *C. officinalis* biosorption of MG dye is compatible with Freundlich and Langmuir isotherms, indicating efficient adsorption in various situations.

### Biosorption isotherms

**Figure 9:**
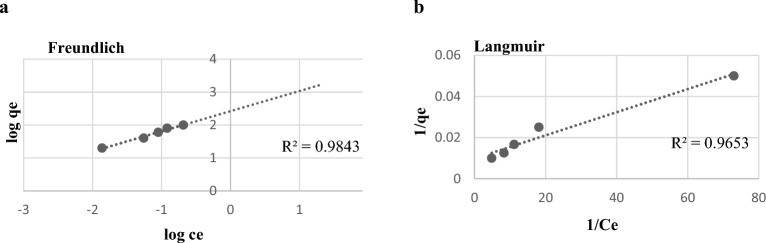
Freundlich (**a**) and Langmuir (**b**) isotherm models for the MG biosorption pH: 6, algae biomass: 30 mg/L, time: 10h.

Adsorption isotherms describe the relationship between the adsorbent and the analyte concentration in the solution^24^. At optimal conditions, the isotherms models for MG biosorption on dried *Corallina officinalis* biosorbent were used. Figure 9. shows the biosorption Freundlich (Fig. 9a) and Langmuir (Fig. 9b) isotherms correlations which were calculated to be 0.9843 and 0.9653, respectively, and their constants are listed in Table 2. The biosorption was compatible with both the Freundlich and Langmuir isotherms models, demonstrating that MG dye is effectively adsorbed on *C. officinalis* biomass, and the biomass may have the highest binding affinity and maximum capacity for MG dye and may display both heterogeneous (multilayer adsorption) surface conditions and homogenous adsorption (monolayer adsorption)^25^. According to Siew Ling (2011)^26^, for defining the dye sorption by the red alga, strong correlation coefficient values for the Langmuir and Freundlich models (R2 > 0.95) are preferred, which is compatible with our findings.

**Table 2:** Freundlich and Langmuir constants for MG biosorption on dried *Corallina officinalis*.

Freundlich			Langmuir		
K_F_	1/n	R^2^	q_max_ (mg g^−1^)	K_L_ (1 mg^−1^)	R^2^
264.3053	0.611	0.9843	101.30	17.54	0.9653

The primary goal of water reclamation was to reuse and conserve water. However, relatively little consideration is paid to the consequences of wastewater components on plants, animals, the environment, and human health. Proper rules and guidelines are required to limit the reuse of various types of industry treated water. *Corallina officinalis* biosorbent usage may have ecological impacts, disrupting marine ecosystem balance and biodiversity. Unmanaged harvesting practices may cause unforeseen environmental consequences, including interactions with organisms, by-product release, and long-term effects on water quality and ecosystem dynamics.

### Biological activities of *Corallina officinalis*

#### Antioxidant activity

The scavenging of the free radical scavenging activity of samples was measured by 1,1-diphenyl-2-picryl-hydrazil (DPPH), superoxide anion radical, and 2,2-Azino-bis (3-ethylbenzothiazoline-6-sulfonic acid) diammonium salt (ABTS) radicals of the studied *Corallina officinalis* algal extract are represented in Table 3. *C. officinalis* algal extract exerted significant radical scavenging activity against free radicals (DPPH, superoxide anion, and ABTS) showing dose-dependent percent inhibition. The ability of the macroalgal extract to scavenge DPPH, superoxide anion, and ABTS increased with the increase of algal extract concentration. Values are expressed as mean + SD of three measurements.

**Table 3:** Antioxidant activity of acetone extract of *Corallina officinalis* extract.

Antioxidant assay (%)	Extract concentration (µg ml^−1^)		
	25	50	75
DPPH radical scavenging	18.65 ± 0.46	54.09 ± 0.67	66.75 ± 0.46
Superoxide anion scavenging	12.90 ± 0.61	37.64 ± 0.42	49.82 ± 0.40
ABTS radical scavenging	29.42 ± 0.57	46.39 ± 0.63	78.26 ± 0.85

Values are expressed as mean + SD of three measurements.

Algae are recognized as important antioxidants because of their capacity to scavenge free radicals such singlet oxygen, superoxide, and hydroxyl radicals^27^. Using different antioxidant bioassays, the algal extracts that were tested showed moderate antioxidant activity. The DPPH test was used as the primary evaluation method, being considered a reliable, simple, and widely used colorimetric method for pre-screening new antioxidants from natural sources^28^. The other two used methods helped to further understand the mechanism of antioxidant activity.

Previous research has reported strong antioxidant activity in the algae *Portieria hornemannii* through using different extraction solvents such as ethyl acetate, methanol, and chloroform^29^. In contrast to previous findings, this study discovered that *C. officinalis* acetone extracts showed comparatively strong antioxidant activity, probably because the extracts included more antioxidant components^27^. This raises the possibility of Corallina alga being used as a natural source of antioxidants, possibly in place of synthetic antioxidants like butylated hydroxyanisole and hydroxytoluydrotoluene, which are known to cause cancer when present in high amounts^30^.

#### Antimicrobial activity

The antibacterial efficacy of *Corallina officinalis* extracts against gram-positive and gram-negative bacterial strains is displayed in Table 4. The extract inhibited all of the tested microorganisms at relatively low concentrations, with significant minimum inhibitory concentration (MIC) values of 0.156 mg mL^−1^ for *Bacilus. mycoides* and 1.25 mg mL^−1^ for *Candida albicans*. MIC values for fungi ranged from 1.25 to 5 mg mL^−1^. Although the tested extract had weaker activity than standard antibiotics, it demonstrated more potent antibacterial than antifungal properties, consistent with previous studies that attributed differences in cell wall permeability and structure to gram-positive and gram-negative bacteria and fungi sensitivity^31^. Compared to gram-negative bacteria, gram-positive bacteria were just slightly more sensitive, perhaps as a result of the latter's hydrophilic cell wall structure^32^. Chitin and glucan are two examples of the polysaccharides that make up the fungal cell wall, which make it less permeable^33^ .

**Table 4:** MIC of acetone extract of *C. officinalis*.

Microorganisms	*Corallina officinalis*	DMSO	S	K
*E. coli*	1.25	na	0.03125	–
*S. aureus*	0.625	na	0.03125	–
*B. subtilis*	0.156	na	0.00781	–
*B. mycoides*	0.156	na	0.00781	–
*K. pneumonia*	1.25	na	0.00195	–
*E. cloaceae*	0.312	na	0.00195	–
*A. flavus*	2.5	na	–	0.0039
*A. fumigatus*	2.5	na	–	0.0039
*M. mucedo*	5	na	–	0.03125
*C. albicans*	1.25	na	–	0.00195
*B. cinerea*	5	na	–	0.00195
*T. harzianum*	2.5	na	–	0.00781

MIC values are given for the tested sample and antibiotics in mg mL^−1^.

*K* ketoconazole, *S* streptomycin, *na* not active.

#### Cytotoxicity activity

The substantial cytotoxic activity of *Corallina officinalis* against the examined cancer cell lines in vitro is displayed in Table 5. Higher cytotoxic activity corresponds to a lower IC_50_*. C. officinalis* was found to be significantly cytotoxic to HCT-15 cell line, whereas IC_50_ was 25.895 µg mL^−1^. Previous studies have indicated that the anticancer effects are due to components found in algae. However, determining how each component contributes to the overall anticancer properties is challenging. The extract’s activities are frequently the outcome of many substances' synergistic interactions. Only a few reports to date have indicated that *Corallina officinalis* has anticancer properties in the literature^34^. Kwon et al.^35^ reported that *Corallina pilulifera* inhibited the growth of the HeLa cell line with a correlation between our results. Our findings indicated that the tested alga had significantly moderated anticancer activity when compared to their findings.

**Table 5:** Growth inhibitory effect of acetone extract of *C. officinalis*.

Cancer cell lines	*Corallina officinalis* IC_50_ (μg mL^−1^)
A549	43.075 ± 0.431
HCT-15	25.895 ± 0.289
MG-63	39.48 ± 0.551
PC-3	46.87 ± 0.933
MCF7	55.3 ± 0.028
Hela	77.775 ± 0.799
Vero	90.09 ± 0.367
L6	115.2 ± 0.325
Fem-x	87.09 ± 0.608

Values are expressed as mean ± SD of three parallel measurements.

The study emphasizes *Corallina officinalis'* possible health advantages. *C. officinalis* acetone extract has antioxidant, cytotoxic, antibacterial, and antifungal properties. These discoveries pave the way for further research and development of medicines and bioactive chemicals.

### Contribution of macroalgae in achieving sustainable development objectives (SDGs)

To ensure that the world has a bright future, the United Nations (UN) added the SDGs to the 2030 Agenda for Sustainable Development. At first glance, these goals may seem hard to understand, but they explain a number of societal problems and give a detailed list of steps for improving how people interact with their environment. In 2015, the global community released the 2030 Agenda, which lists the 17 SDGs that must be met by 2030. If you do not have access to clean water and basic sanitation services, your health is at risk. Additionally, it is a barrier to growth for a sizable portion of the global populace, particularly the most vulnerable^6^.

Because of the multiple roles that macroalgae play in aquatic ecosystems and society, they can be thought of as an effective weapon in the fight to achieve the SDGs. Macroalgae play a big role in the ecology of coastal and oceanic habitats because they create unique nursery areas that are important to the environment and remove inorganic pollutants, which helps clean up the water.

In this study, we look at and talk about how macroalgae might help the UN's Sustainable Development Goals. We focus on SDG 3, which is about good health; SDG 6, which is about improving water quality; and SDG 13, which is about fighting climate change^36^.

Based on what we found, *Corallina officinalis* extracts can be used as food supplements and as a natural source of antioxidant, antibacterial, and anticancer drugs. These health benefits contribute to the achievement of SDG 3. *C. officinalis* can be used to get rid of toxic dyes in industrial wastewater that are bad for people's health and the environment, which will help reach SDG 6. *Corallina officinalis* can take in malachite green dye and lock it away in its cell walls. With this technology, wastewater dye pollution from industrial companies can be cut in a way that is cheap, uses less energy, and is good for the environment. *Corallina officinalis* can cut down on pollution and act as living protection structures, which increases the value of the environment and helps reach SDG 13.

The research focuses on the elimination of malachite green dye and the bioactivities of *Corallina officinalis*, but its relevance to different dyes, pollutants, or toxins may vary. Further investigation is needed to determine its efficacy for different chemicals. Environmental considerations and regulatory and commercial considerations are crucial for successful commercialization.

## Conclusion

The finding of the current study showed that *Corallina officinalis* is an efficient, affordable, and eco-friendly biosorbent for eliminating toxic malachite green dye from aqueous solutions. The removal efficacy of dye reached up to 99.9% and maximum adsorption capacity 101.30 mg g^−1^ at specified conditions: pH 6, 27 °C, 120 rpm shaking at 20 mg L^−1^ initial dye concentration, and 0.03 g L^−1^ of biomass at contact time 2 h. Moreover, the biosorption of MG was well fit for the pseudo-second-order model with linear regression correlation coefficient ${r}_{2}^{2}$ values with an average of 0.95232. It was compatible with both the Freundlich and Langmuir isotherms models demonstrating that MG dye is effectively adsorbed on *C. officinalis* biomass, and the biomass may have the highest binding affinity and maximum capacity for MG dye and may display both heterogeneous surface conditions and homogenous adsorption. This would serve as an example of the utilization of marine resource in water treatment technology to reduce environmental contamination with numerous promising advantages for future commercial use, cut down on pollution and they can be thought of as an effective weapon in the fight to achieve the SDGs.

According to our findings, the acetone extract of *C. officinalis* exhibits significant an antioxidant for ABTS radical scavenging with 78.26%, significant cytotoxic activity against Colon adenocarcinoma (HCT-15) with IC_50_ 25.895 µg mL^−1^, remarkable antibacterial activity against *B. mycoides, B. subtilis* with MIC 0.156 mg mL^−1^, and significant antifungal activity against *candida albicans* with MIC 1.25 mg mL^−1^. Human health systems are currently concerned about hazardous germs becoming resistant to synthetic antibiotics, so it is crucial to think of creative remedies. *Corallina officinalis* is an abundant source of pharmaceuticals and bioactive substances that can satisfy the population’s expanding needs.

### Future work

The current study is the first scientific examination of Corallina officinalis for its ability to remove malachite green dye. The current study proved *C. officinalis's* adsorption efficacy, and its biological activities have been found. These findings could provide valuable information for future efforts to remove additional dyes and heavy metals. More research is needed to assess the nutritional characteristics and mechanisms underlying the health benefits of various macroalgal products.

## Materials and methods

### Collection of algae

At low tide, samples were collected from locations along Egypt's Red Sea shorelines. To remove salt, sand, and epiphytes, samples were washed in distilled water before being air dried at room temperature. and subsequently dried for 24 h in an oven at 60 °C. A powdered form of dried samples was sieved and kept at room temperature in tight dark vials until testing^37^.

### Genomic isolation and purification

The internal transcribed spacer (ITS) molecular marker was employed in this study to distinguish the algae samples. The NanoMag Plant and Algae DNA Isolation System (Attogene, USA) was employed. To isolate complete DNA from plants and algae, the NanoMag Plant and Algae DNA Isolation Kit NA2012-01 was specifically created. It is crucial to make sure that the sample pretreatment procedure is carried out at a low temperature because it will directly impact the creation of DNA and the integrity of the fragments. The proprietary magnetic beads and carefully formulated buffer are included in the NanoMag Plant and Algae DNA Isolation Kit. The successfully eluted purified DNA can then be stored at a temperature of −20 °C or used in PCR or other enzymatic reactions. On the magnetic particle processor instrument, the procedures are simple to use and can be completely automated. A CreaCon heat cycler was used to perform Amplicon (Holand). The length of the finished amplified product was calculated using the DNA ladder 1kbp DNA marker (PeqGold 1 Kb, Peqlab, and GMH). The COI gene was amplified using PCR using the recovered DNA. Table 6 lists the primers used for PCR and sequencing. Sequencing and PCR were performed utilizing the techniques outlined by Kogame et al.^38^ .

**Table 6:** Specific primer sequence under study.

Primers	Sequences		Target bp	References
	COICorF1	(5′ TCCTCTAAGTTCAATACAAAG 3′)	624 bp to 664 bp	Kogame et al*.*, 2017^38^
	COICorR1	(5′ AAGCTCCTGCTATATGTAAA 3′)		
	COICorR2	(5′ GAYCAYACAAATAAYGGWATTC 3′)		

### Specific gene detection

Green taq (DreamTaq) master mix (Thermo scientific) was used for gene amplification according to manufacture protocol**.** According to Kogame et al. (2015)^38^, thermal cycler conditions applied as follow, 10  min at 96 °C for denaturation, followed by 40–50 cycles of 30  s at 94 °C, 30 s at 50 °C and 30  s at 72 °C, with a final extension of 5  min at 72 °C. Final product for specific amplicon was photograph and detection using Dig-doc, UVP, INC, England.

### Electrophoresis CONDITIONS

PCR products were loaded on 1.5% (w/v) Agarose gel, stained with Ethidium bromide, separated by electrophoresis (75 V, 150 mA) and viewed on UV plate. Gene JET PCR Purification Kit (Thermo Scientific) was used for DNA purification. ABI PRISM^®^ 3100 Genetic Analyzer was applied for PCR products and performed by Macrogen In. Seal, Korea.

### Data analysis

Gel documentation system (Geldoc-it, UVP, England), was applied for data analysis using Totallab analysis software, ww.totallab.com, (Ver.1.0.1). Positive amplicons were eluted from agarose gel through E.Z.N.A.^®^ Gel Extraction Kit (V-spin) (Omega BIO-TEK). Sequence analysis was employed using the ABI PRISM^®^ 3100 Genetic Analyzer (Micron-Corp. Korea).

### Spectral analysis

FTIR was performed to investigate the adsorbent surface’s functional groups that are in charge for the adsorption process within the range of 4000–400 cm^−1^^39^.

### Scanning electron microscopy

SEM was used to investigate the morphology of dried *C. officinalis* powder before and after the MG biosorption process^39^.

### Preparation of MG solution

MG is a cationic dye with molecular weight MW: 972.02 g mol^−1^ supplied by Research-Lab Fine Chem Industries, India. Weighted amounts of MG were dissolved in 1 L of distilled water to prepare the required concentrations of fresh stock solution C, with batches of 20, 40, 50, 60, 80, and 100 mg L^−1^^40^. All chemical reagents are analytical research grade (AR).

### Batch biosorption experiments

In a 50 mL Erlenmeyer beaker, all biosorption tests were conducted for 10 h with constant swirling at 120 rpm and a room temperature of 27 °C. A Hettich, EBA 200 centrifuge was used to centrifuge the solution after it had reached balance for 10 min at 3000 rpm. It was then examined. By measuring the absorbance at a wavelength of 618 nm, an Ultra-violet visible spectrophotometer (UV–VIS spectrophotometer T80+) was used to quantify the uptake dye^41^.

The effect of pH was assessed using 0.03 g L^−1^ of *Corallina officinalis* and 30 mL of initial MG concentration 50 mg L^−1^ at different solution pH from 2 to 8. The pH of the solution was changed by adding 0.1 N NaOH or HCL solution using a pH meter to examine the influence of pH on sorption capacity^42^.

Various doses of biosorbent were used: 0.02, 0.03, 0.05, 0.06, and 0.08 g of algae with 30 mL of MG of initial dye concentration 100 mg L^−1^ to examine how biosorbent affects dye removal at ideal solution pH.

Different dye concentrations, from 20 to 100 mg L^−1^, were used to examine the effect of the initial dye concentration on dye removal by using 0.03 g L^−1^ of algae and 30 mL of MG in a series of the beaker at room temperature 27 °C for 10 h. The solution was centrifuged after reaching equilibrium for 10 min at 3000 rpm using a Hettich, EBA 200 centrifuge. Then, it was analyzed. The uptake dye was measured by a UV–VIS spectrophotometer (T80+) by examining the absorbance at a 618 nm wavelength^41^. The results represent the two replicates average values.

Equilibrium is an essential requirement for the use of an adsorption technique in the treatment of wastewater. To investigate the effect of contact time on MG dye sorption, 0.03 g L^−1^ biomass of *Corallina officinalis* was put into contact with 30 mL of each concentration of dye (20, 40, 60, 80, and 100 mg L^−1^) at pH 6 for 10 h at 120 rpm and room temperature 27 °C.

A kinetics study was performed at optimum pH. A series of 50 mL beakers containing 0.03 g L^−1^ of algae and 30 mL of MG at initial concentrations of 20, 40, 60, 80, and 100 mg L^−1^ were shaken at a constant speed while performing the kinetics studies. The samples were obtained at regular intervals^43^. The biosorption kinetics of dye by biosorbent was explained using the pseudo-first-order and pseudo-second-order models. The amount of MG adsorbed onto *Corallina* o*fficinalis* and the removal efficiency (R %) of MG were calculated using Eqs. (7) and (8), respectively^44^. In these equations, q_e_ (mg g^−1^) represents adsorption capacity, *C*_*i*_ is the initial concentration of MG at time 0, and *C*_*f*_  is the final concentration of MG mg L^−1^. At time t, *V*  is the volume of dye solution in L and *M*  is the mass of adsorbent in g:

7 $${\text{Adsorption capacity }}\left( {{\text{mg g}}^{{ - {1}}} } \right) \, = \, (Ci - Cf) \times {\text{V}}/{\text{M}}$$

8 $${\text{R }}\% \, = Ci - Cf/Ci \times { 1}00$$

### Biosorption isotherms

The most commonly used biosorption isotherm models are the Langmuir and Freundlich models. They were determined in this study using experimental data. Equations (9) and (10) for the isotherm model's calculations were as follows^45^:

Langmuir isotherm equation

9 $$= \frac{Ce }{qe} = \frac{1}{qm Kl}+\frac{Ce}{qm}$$

Freundlich isotherm equation

10 $${\text{lnq}}_{{\text{e}}} = {\text{ ln}}K_{f} + {\text{ lnCe}}$$

C_e_ is the concentration of dye ion at equilibrium (mg L^−1^), q_m_ is the maximum capacity of adsorption (mg g^−1^), K_L_ is the Langmuir isotherm constant (L mg^−1^), K_F_ is the Freundlich isotherm constant (L mg^−1^).

### Investigation of the biological activity of *Corallina officinalis*

#### Preparation of the algae extract

Examined algae that had been finely ground desiccated (100 g) were extracted with acetone (500 mL). Before being condensed in a rotary evaporator at low pressure, the extracts were filtered. Before being used in the experiments, the dry extracts were stored at −18 °C. The samples were then dissolved in 5% dimethyl sulfoxide (DMSO), which was used as a solvent control test to look into DMSO's impact on the development of microorganisms^27^.

#### Antioxidant activity

 The various mechanisms by which antioxidants work, the antioxidant potential of *Corallina officinalis* was evaluated using three assays: the DPPH radical scavenging activity, ABTS radical, and superoxide anion scavenging activity. DPPH was used to assess the samples' capacity to scavenge free radicals . The experiment was performed in triplicates. The capacity of the *C. officinalis* algal extract to scavenge free radicals was evaluated using the (DPPH), superoxide anion, (ABTS) methods as outlined by Kosanić et al.^27^.

#### Antimicrobial activity

In our research, the following microbes were used as test organisms: *Bacillus mycoides* (ATCC 6462), *Bacilus subtilis* (ATCC 6633), *Staphylococcus aureus* (ATCC 25923), *Escherichia coli* (ATCC 25922), *Klebsiella pneumoniae* (ATCC 33495), and *Enterobacter cloaceae* (ATCC 13047). Additionally, the used fungi were *Aspergillus flavus* (ATCC 9170), *Aspergillus fumigatus* (ATCC 1022), *Candida albicans* (ATCC 10231), *Mucor mucedo* (ATCC 52568), *Botrytis cinerea* (ATCC 36634), and *Trichoderma harzianum* (ATCC 20476).

Human and fish bacterial and fungal pathogens were obtained from the Naval Medical Research Unit 3, Cairo, Egypt. Bacterial cultures were grown on agar dishes. Potato dextrose (PD) agar and Sabourad dextrose (SD) agar were used to sustain fungal cultures, and fresh mature cultures grown on PD agar at 30 °C were used to make fungal spore suspensions. According to Espinel-Ingroff's approach^46^, in order to measure turbidity spectrophotometrically at 530 nm, spores were rinsed with clean, distillate water. They were then attenuated to a concentration of about 10^6^ CFU mL^−1^.

Bacterial inocula were created by growing bacterial colonies on Mueller–Hinton agar substrate for 24 h at 37 °C. These inocula were then diluted to the 0.5 McFarland standard, or roughly 10^8^ CFU mL^−1^^47^.

All cultures were subcultured for 15 days at 4 °C. Using the broth microdilution method and 96-well microtitre plates, MIC was determined. Against each of the microorganisms tested in the experiment, extract doses varying from 50 to 0.195 mg mL^−1^ were used. The original extract solutions were made by measuring off a specific amount of extract and mixing it with DMSO. For bacterial colonies, use the Muller-Hinton broth; for fungi, use the SD broth, extracts were produced in twofold dilutions.

Ketoconazole and streptomycin were the standards for fungi and bacteria, respectively. To find out how 5% DMSO affected the development of a bacterium, the solvent control test was conducted. By determining MIC, the susceptibility of microorganisms to acetone extracts of the studied algae was evaluated, where the MIC was determined using Resazurin^31^.

### Cytotoxic activity

#### Cell culture

Cancer cell lines in this research were human lung carcinoma (A549), human melanoma (Fem-x), colon adenocarcinoma (HCT-15)**,** human osteosarcoma (MG-63), prostate carcinoma (PC-3), human breast adenocarcinoma (MCF-7), HeLa, African green monkey kidney (Vero), and rat skeletal muscle myoblast (L6) cell lines. Cell lines were obtained from the Naval Medical Research Unit 3, Cairo, Egypt. Cells were cultured in a monolayer at 37 °C in a humidified air environment of 95% and 5% CO_2_ in the RPMI 1640 nutrient medium with 10% (inactivated at 56 °C) fetal bovine serum (FBS), 3 mM of l-glutamine, and antibiotics^31^.

#### In vitro cytotoxic assay

The tested extract was in vitro tested for cytotoxic action. The required working amounts of the extract stock solution (50 mg mL^−1^) were dissolved in the appropriate medium. A549 cells (5000 cells/well), Fem-x cells (5000 cells/well), L6 cells (2000 cells/well), HCT-15 cells (4000 cells/well), MG-63 cells (3000 cells/well), PC-3 cells (5000 cells/well), MCF-7, Vero, and HeLa cells (roughly 2104 cells/well) were all inoculated into 96-well microtitre plates^48,49^. Except for the control cells, which simply received a nutritional medium addition, The wells received five varying concentrations of the examined extracts 24 h after cell adhesion. Final concentrations were 200, 100, 50, 25, and 12.5 g mL^−1^ in the treatment wells. After that, the colonies were incubated for 72 h.

The impact on cancer cell survival was evaluated using the 3-(4,5-dimethylthiazol-2-yl)-2,5-diphenyltetrazolium bromide (MTT) assay 72 h after the addition of the extract. Briefly, 20 µL of MTT solution (5 mg mL^−1^ Phosphate Buffered Saline (PBS) were added to each well, and they were then kept for an additional 4 h at 37 °C in 5% CO_2_ and humidified air. The formazan crystals made from MTT were then solubilized with 100 L of 10% Sodium Dodecyl Sulfate (SDS) following their conversion by the mitochondrial dehydrogenases of live cells. A microplate reader (Multiskan EX, Thermo Scientific, Finland) was used to measure the absorbencies at 570 nm, which were proportional to the number of live cells. The cells were not harmful since the DMSO solvent's final concentration was never more than 0.5%. The term "IC_50_ concentration" refers to the amount of a substance needed to prevent 50% of cells from surviving. The results represent the three replicates average values (Supplementary Information).

### Supplementary Information

Supplementary Information.

## Data Availability

The datasets used and/or analyzed during the current study available from the corresponding author on reasonable request.
