# Supplementary material for: Biological activities and biosorption potential of red algae (Corallina officinalis) to remove toxic malachite green dye
Source: Sci Rep. 2023 Aug 24;13:13836. doi: 10.1038/s41598-023-40667-8 (PMC10449875; doi:10.1038/s41598-023-40667-8)
Supplement: Supplementary file 1 — Supplementary Information. [file 41598_2023_40667_MOESM1_ESM.docx]

**Biological Activities and Biosorption Potential of Red Algae (*Corallina Officinalis*) to Remove Toxic Malachite Green Dye**

Elen Emad Youssef^1*^, Botros Beshay^1^, Kareem Tonbol^2^, and Sarah Makled^3^

1. **DNA Identification and Gene Isolation**

1.1 Specific genomic product for ITS1 and 2 with ≈ 265 bp

| a | b | c |
| --- | --- | --- |
| 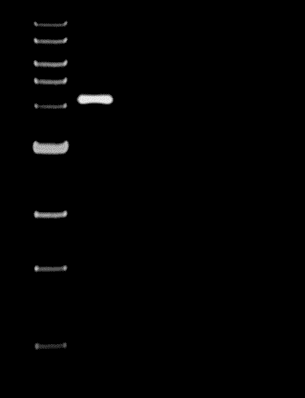 | 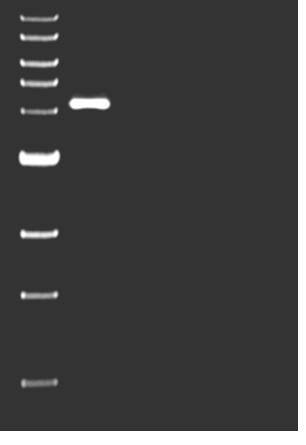 | 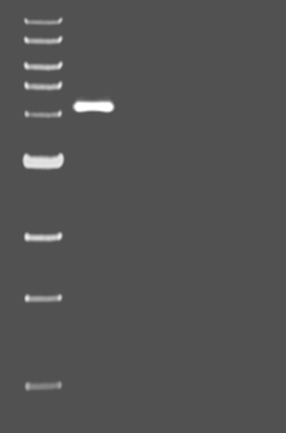 |

| d | e | f |
| --- | --- | --- |
| 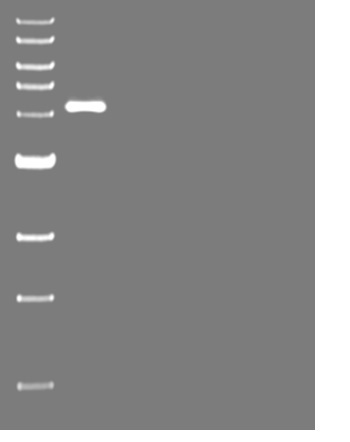 | 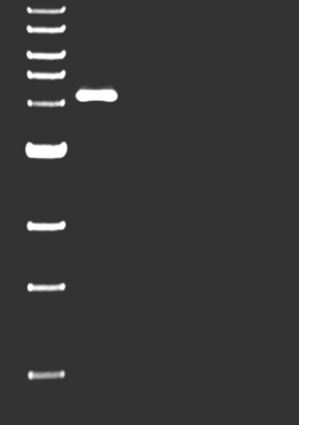 | 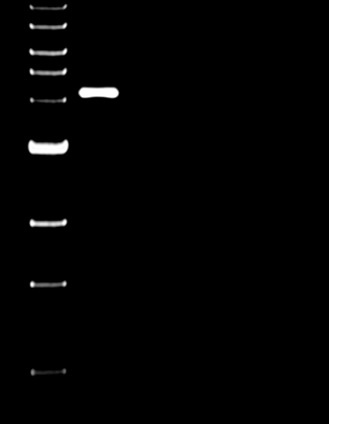 |

Loaded amplicon on Agarose gel image was documented via Gel documentation system (Geldoc-it, UVP, England) integrated with Totallab analysis software, ww.totallab.com, (Ver.1.0.1) which used for data interpretation. Gel interested area was automatically detected and identified through Gel documentation system (Geldoc-it, UVP, England). Also, image contrast and brightness were fully computerized and controlled via Totallab analysis software, ww.totallab.com, (Ver.1.0.1). Manual images contrast alignment was included.

2.1 Computerized detection Specific genomic product for ITS1 and 2 with ≈ 265 bp


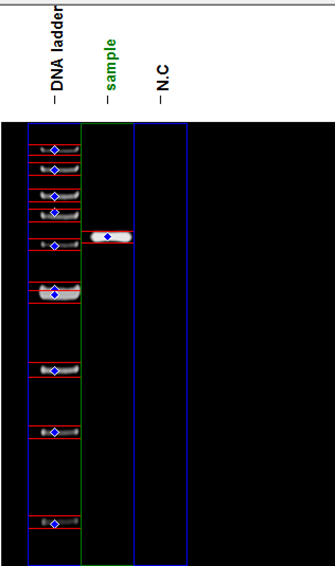


3.1 Computerized fragments length detection for Specific genomic product for ITS1 and 2 with ≈ 265 bp.


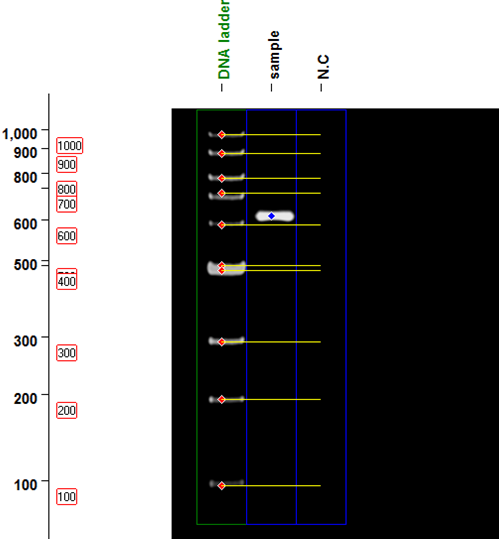


1. **Data analysis for DNA ladder**

**
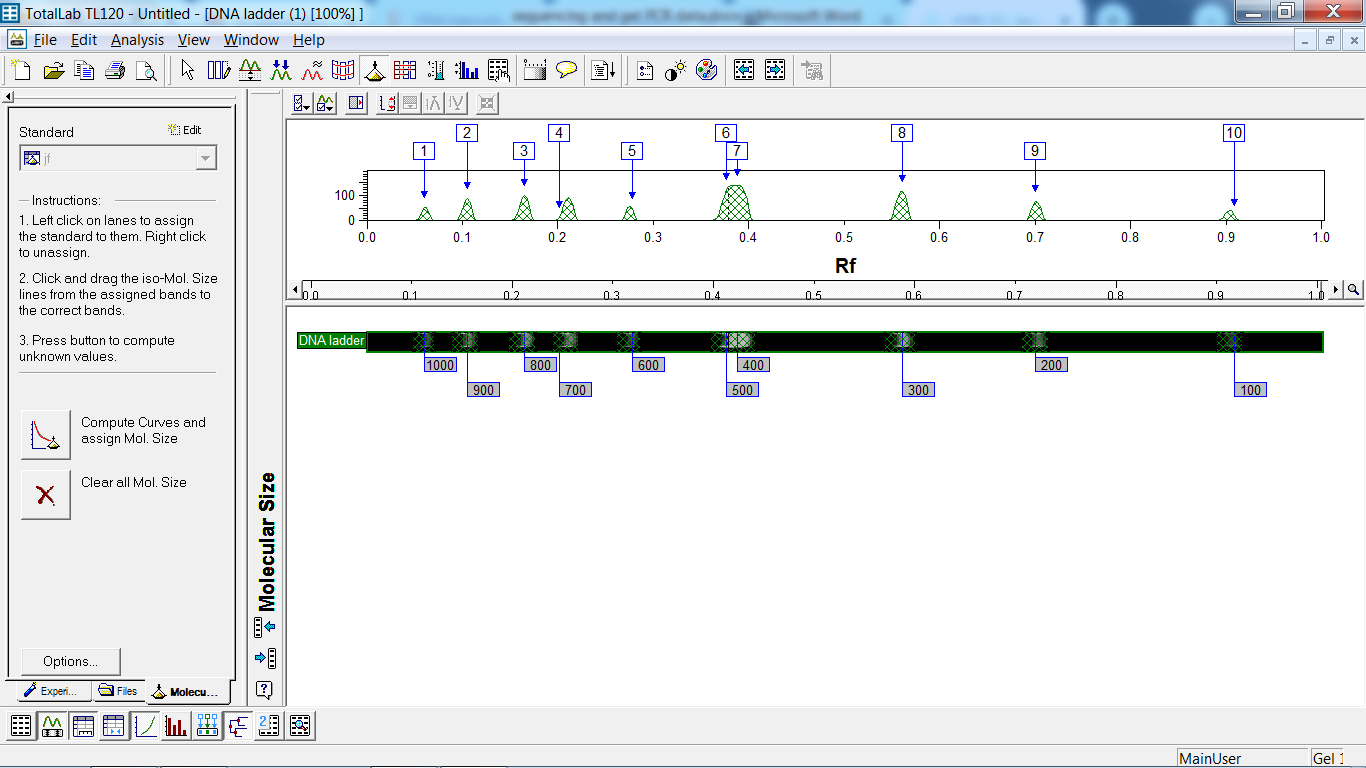
**

**
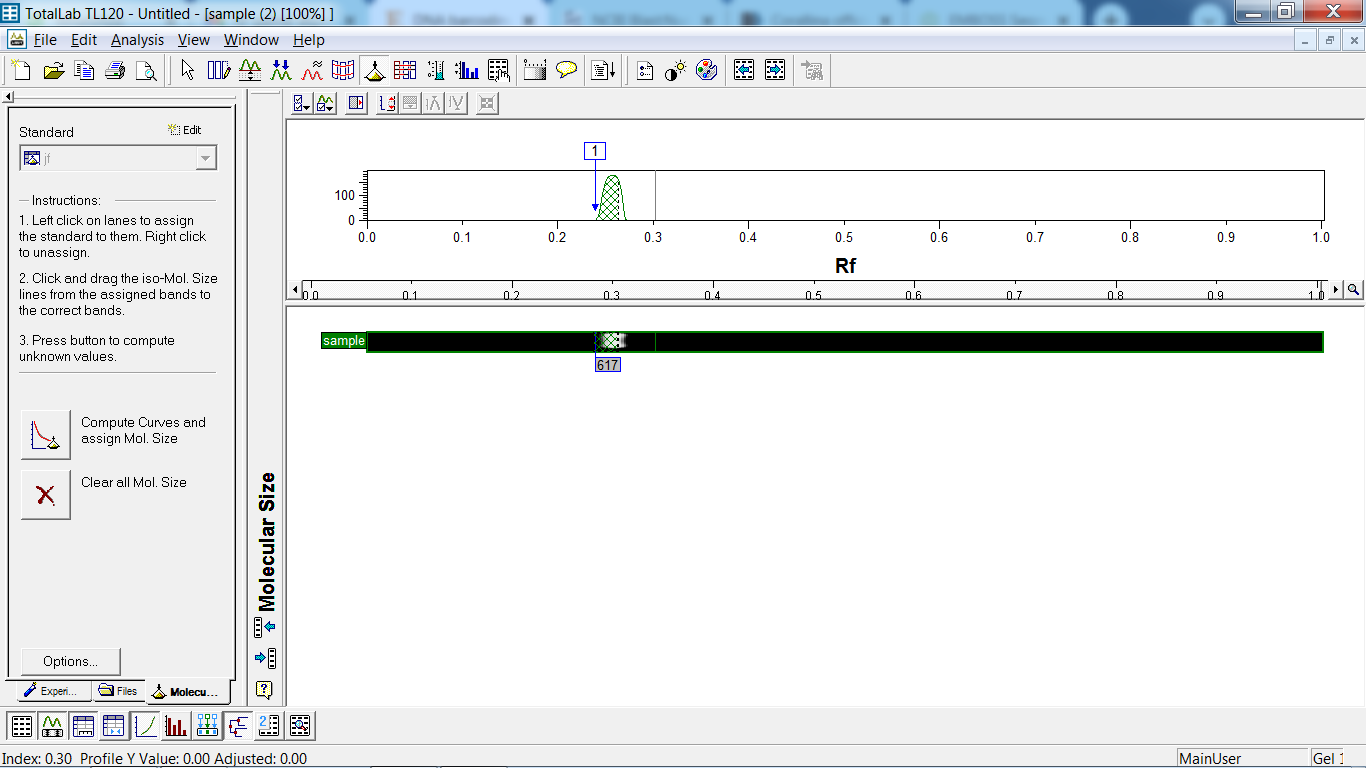
**

1. **Data parameters for DNA ladder and samples**


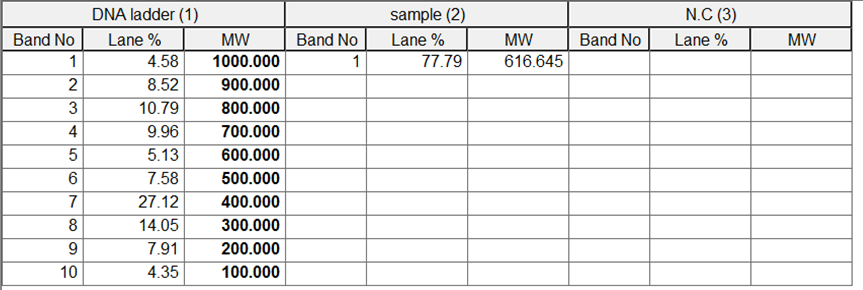


1. **Methods**

In this investigation, ITS molecular marker was used to identify algae samples.

**Genomic isolation and purification:**

NanoMag Plant and Algae DNA Isolation Kit Catalog Number:NA2012-01 (Attogene, USA) was used. NanoMag Plant and Algae DNA Isolation Kit NA2012-01 is specially designed for purification of total DNA from Plants and Algae. The pretreatment method of plant samples will directly affect the production of DNA, the integrity of the fragments thus it is important to ensure that the sample pretreatment process is performed at low temperature.  The NanoMag Plant and Algae DNA Isolation Kit comes with proprietary magnetic beads and a specially formulated buffer. The purified DNA can then be effectively eluted with EL and will be ready for use in PCR or other enzymatic reactions or storage at -20°C. The procedures can be fully automated on the magnetic particle processor instrument and ease of use. Amplicon was carried out using a CreaCon thermal cycler (Holand). DNA ladder 1kbp DNA marker (PeqGold 1Kb, Peqlab, GMH) was used to estimate final amplified product length.

**Specific gene detection**

Green taq (DreamTaq) master mix (Thermo scientific) was used for gene amplification according to manufacture protocol**.** According to Kogame et al., (2015), thermal cycler conditions applied as follow, 10 min at 96°C for denaturation, followed by 40–50 cycles of 30 s at 94°C, 30 s at 50°C and 30 s at 72°C, with a final extension of 5 min at 72°C. Final product for specific amplicon was photograph and detection using Dig-doc, UVP, INC, England.

**Electrophoresis conditions:**

PCR products were loaded on 1.5% (w/v) Agarose gel, stained with Ethidium bromide, separated by electrophoresis (75 V, 150 mA) and viewed on UV plate. Gene JET PCR Purification Kit (Thermo Scientific) was used for DNA purification. ABI PRISM® 3100 Genetic Analyzer was applied for PCR products and performed by Macrogen In. Seal, Korea.

**Data analysis:**

Gel documentation system (Geldoc-it, UVP, England), was applied for data analysis using Totallab analysis software, ww.totallab.com, (Ver.1.0.1). Positive amplicons were eluted from agarose gel through E.Z.N.A.® Gel Extraction Kit (V-spin) (Omega BIO-TEK). Sequence analysis was employed using the ABI PRISM® 3100 Genetic Analyzer (Micron-Corp. Korea).

1. **Specific Primer sequence under study**

| Primers | **Sequences** | | **Target bp** | **References** |
| --- | --- | --- | --- | --- |
|  | **COICorF1** | (5′ TCCTCTAAGTTCAATACAAAG 3′) | 624 bp to 664 bp | Kogame *et al.*, 2017 |
|  | **COICorR1** | (5′ AAGCTCCTGCTATATGTAAA 3′) |  |  |
|  | **COICorR2** | (5′ GAYCAYACAAATAAYGGWATTC 3′) |  |  |

1. **Master Mix component for PCR reactions**

| **Master Mix component** | **Amount** | **Final concentration** |
| --- | --- | --- |
| Sterile nuclease free water | 23 µl |  |
| **Green taq (DreamTaq) master mix** | 25 µl | 1.0 x |
| Primer (5pmol /µl) | 1.0 µl | 5.0 pmol |
| DNA extracted sample (50ng /µl) | 1.0 µl | 25.0 ng |
| Total | 50.0 µl |  |


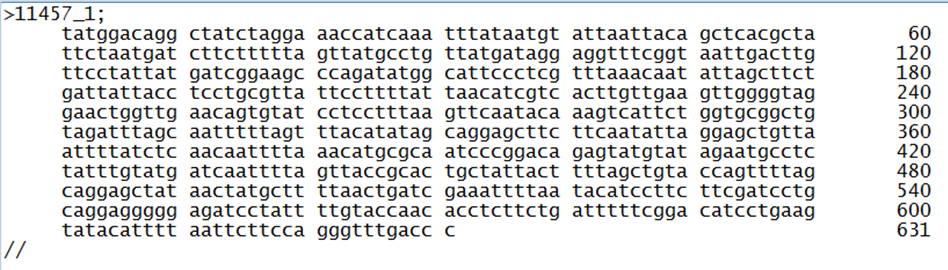


1. **Allingemented Data**


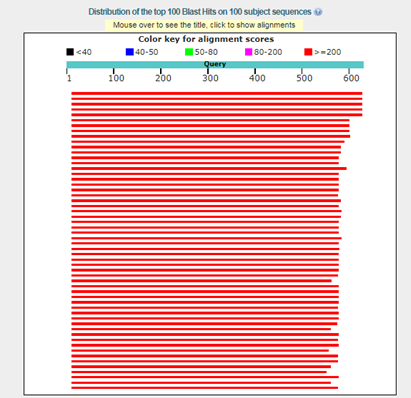


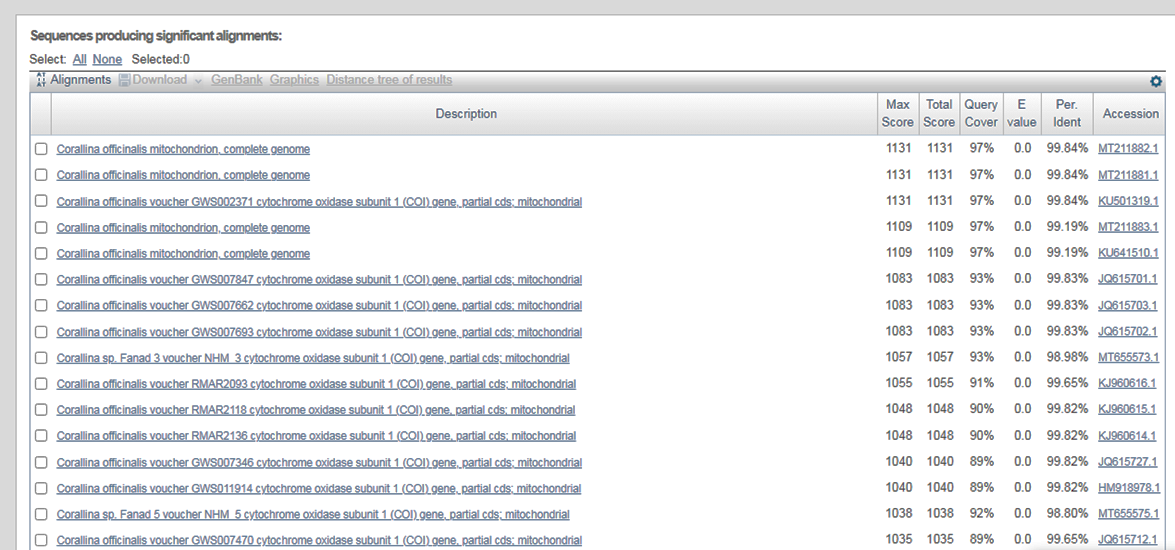


1. **Identification Data for Algae Sample**

| Accession number | Highest homology | Identity % |
| --- | --- | --- |
| KU501319.1 | ***Corallina officinalis voucher GWS002371***  ***cytochrome oxidase subunit 1 (COI) gene, partial cds; mitochondrial*** | 99.84 |

References

1. Kogame, K. *et al.* Delimitation of cryptic species of the Scytosiphon lomentaria complex (Scytosiphonaceae, Phaeophyceae) in Japan, based on mitochondrial and nuclear molecular markers. *Phycol. Res.* **63**, 167–177 (2015).

2 . Kogame, K., Uwai, S., Anderson, R. J., Choi, H. G. & Bolton, J. J. DNA barcoding of South African geniculate coralline red algae (Corallinales, Rhodophyta). *South African J. Bot.* **108**, 337–341 (2017).

1. **Statistical Analysis**

Statistical analysis well done by using non-parametric test using Wilcoxon test and data well represented by box plot.

- 1. **Effect of pH**


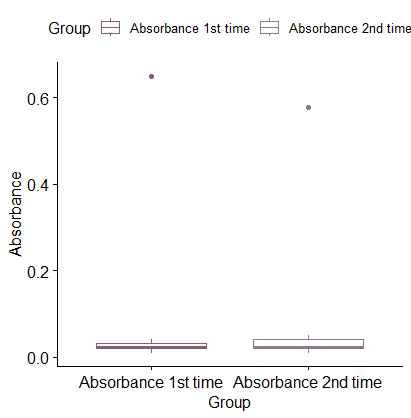


Group count median IQR

1 Absorbance 1st time 7 0.023 0.013

2 Absorbance 2nd time 7 0.023 0.022

Wilcoxon rank sum test:

p-value = 1

The data were represented using box plot. The test showed a non-significant difference owing to the p-value (p> 0.05).

- 1. **Effect of Algae Dose**

**
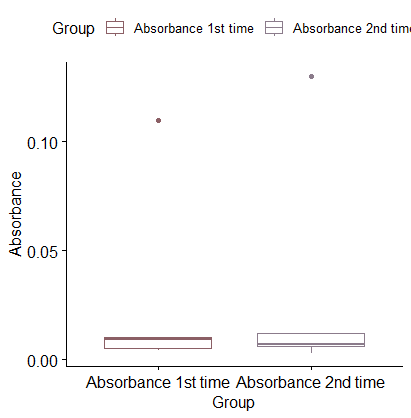
**

Group count median IQR

1 Absorbance 1st time 5 0.009 0.005

2 Absorbance 2nd time 5 0.007 0.006

Wilcoxon rank sum test:

p-value = 1

It was done a non-parametric comparison using Wilcoxon test. The data were represented using box plot. The test showed a non-significant difference owing to the p-value (p> 0.05).

- 1. **Effect of Dye Concentration**


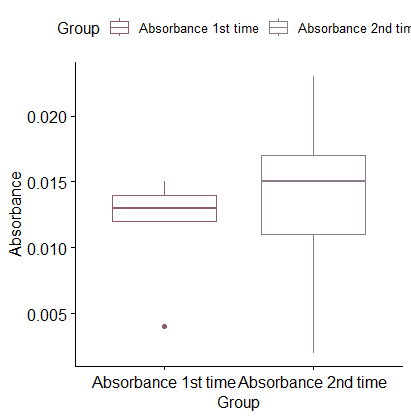


Group count median IQR

1 Absorbance 1st time 5 0.013 0.002

2 Absorbance 2nd time 5 0.015 0.006

Wilcoxon rank sum test:

p-value = 0.6004

It was done a non-parametric comparison using Wilcoxon test. The data were represented using box plot. The test showed a non-significant difference owing to the p-value (p> 0.6).

**2.4 kinetic study at 20 mg /L:**


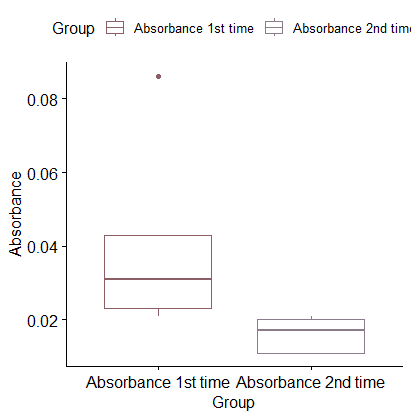


Group count median IQR

1 Absorbance 1st time 5 0.031 0.02

2 Absorbance 2nd time 5 0.017 0.009

Wilcoxon rank sum test:

p-value = 0.01565

**2.5 kinetic study at 40 mg / L:**

**
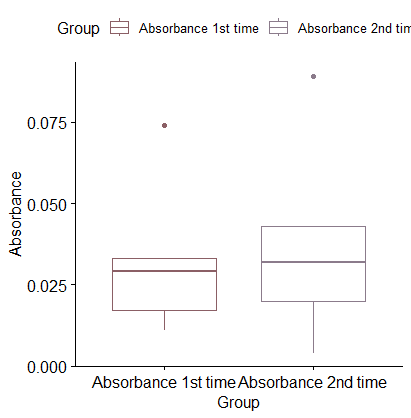
**

Group count median IQR

1 Absorbance 1st time 5 0.029 0.016

2 Absorbance 2nd time 5 0.032 0.023

Wilcoxon rank sum test:

p-value = 0.8345

**2.6 kinetic study at 60 mg / L:**


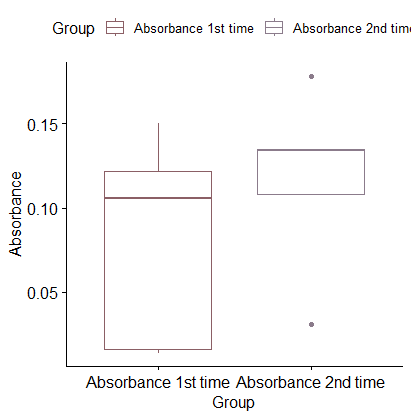


Group count median IQR

1 Absorbance 1st time 5 0.106 0.106

2 Absorbance 2nd time 5 0.134 0.027

Wilcoxon rank sum test:

p-value = 0.2963

**2.7 kinetic study at 80 mg / L:**

**
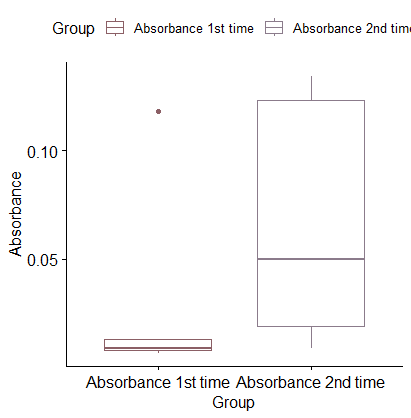
**

Group count median IQR

1 Absorbance 1st time 5 0.009 0.005

2 Absorbance 2nd time 5 0.05 0.104

Wilcoxon rank sum test:

p-value = 0.1161

**2.8 kinetic study at 100 mg / L:**

**
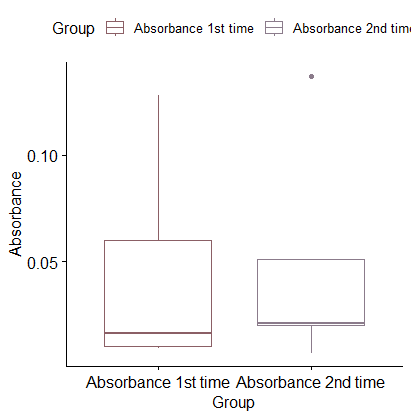
**

Group count median IQR

1 Absorbance 1st time 5 0.016 0.05

2 Absorbance 2nd time 5 0.021 0.031

Wilcoxon rank sum test:

p-value = 0.8345
